# Assessing Mode of Recurrence in Breast Cancer to Identify an Optimised Follow-Up Pathway: 10-Year Institutional Review

**DOI:** 10.1245/s10434-023-13885-7

**Published:** 2023-07-21

**Authors:** Jack Horan, Conor Reid, Michael R. Boland, Gordon R. Daly, Stephen Keelan, Angus J. Lloyd, Eithne Downey, Adam Walmsley, Marie Staunton, Colm Power, Abeeda Butt, Deirdre Duke, Arnold D. K. Hill

**Affiliations:** 1 Department of Breast Surgery, Beaumont Hospital, Dublin 9, Ireland; 2 Department Radiology, Beaumont Hospital, Dublin 9, Ireland; 3 Department of Pathology, Beaumont Hospital, Dublin 9, Ireland; 4 Department of Surgery, Royal College of Surgeons Ireland, Dublin, Ireland

**Keywords:** Breast, Cancer, Recurrence, Surveillance, Mammography

## Abstract

**Background:**

Breast cancer surveillance programmes ensure early identification of recurrence which maximises overall survival. Programmes include annual clinical examination and radiological assessment. There remains debate around the value of annual clinical exam in diagnosing recurrent disease/second primaries. The aim was to assess diagnostic modalities for recurrent breast cancer with a focus on evaluating the role of annual clinical examination.

**Patients and Methods:**

A prospectively maintained database from a symptomatic breast cancer service between 2010–2020 was reviewed. Patients with biopsy-proven recurrence/second breast primary were included. The primary outcome was the diagnostic modality by which recurrences/secondary breast cancers were observed. Diagnostic modalities included (i) self-detection by the patient, (ii) clinical examination by a breast surgeon or (iii) radiological assessment.

**Results:**

A total of 233 patients were identified and, following application of exclusion criteria, a total of 140 patients were included. A total of 65/140 (46%) patients were diagnosed clinically, either by self-detection or clinical examination, while 75/140 (54%) were diagnosed radiologically. A total of 59/65 (91%) of patients clinically diagnosed with recurrence presented to the breast clinic after self-detection of an abnormality. Four (6%) patients had cognitive impairment and recurrence was diagnosed by a carer. Two (3%) patients were diagnosed with recurrence by a breast surgeon at clinical examination. The median time to recurrence in all patients was 48 months (range 2–263 months).

**Conclusion:**

Clinical examination provides little value in diagnosing recurrence (< 5%) and surveillance programmes may benefit from reduced focus on such a modality. Regular radiological assessment and ensuring patients have urgent/easy access to a breast clinic if they develop new symptoms/signs should be the focus of surveillance programmes.

Breast cancer remains the most commonly diagnosed cancer in women worldwide.^1^ The incidence of breast cancer in 2020 was 142.8 cases per 100,000 population in the 27 European Union member states.^2^ Due to the increased use of better and more individualised adjuvant therapies, as well as multidisciplinary management, breast cancer survival has improved over the last decade, with lower rates of recurrence.^3–5^ In addition, recurrences are detected earlier, due to better patient education and improved surveillance modalities.^6^ However, clinicopathological factors such as tumour size, nodal positivity and certain receptor subtypes (TNBC/HER2+ enriched) are still associated with increased rates of recurrence.^7,8^ Recurrent breast cancer or the development of a second primary breast cancer are associated with an increased risk of distant metastatic disease, as well as mortality.^9^ Therefore, diagnosis of recurrence at the earliest possible stage is critical to improving survival.^10,11^

Guidelines from international societies regarding surveillance protocols following a diagnosis of breast cancer are broadly similar in Europe and the USA. Current protocols, involving history and clinical examination combined with interval mammography (MG), are the standard of care. The National Cancer Control Programme (NCCP) in Ireland recommends annual clinical examination for 5 years in addition to annual mammography from diagnosis.^12^ The European Society for Medical Oncology (ESMO) advises history and clinical examination every 3–4 months in the first 2 years following diagnosis, then every 6 months for years 3–5, then annually, with an annual MG.^13^ Guidelines from American societies, including the American Society of Clinical oncology (ASCO) and National Comprehensive Cancer Network (NCCN), are similar and shown in Table 1.^14^ In the UK, some centres offer an open access follow up programme whereby patients undergo annual mammography for 5 years and do not receive routine annual clinical examination. However, patients have access to clinical review if they develop a new symptom or sign. This surveillance method has been shown to be non-inferior to standard follow-up in low-risk patients.^15^

**Table 1:** National societal guidelines for breast cancer surveillance

	Country/continent	History and physical	Mammogram
National Cancer Control Programme	Ireland	6 months for 2 years, then annually for years 3–5	Every 12 months
European Society of Medical Oncology	Europe	3–4 months for 2 years, then every 6 months for 3–5 years, then annually	Every 12 months with ultrasound
American Society of Clinical Oncology	USA	Every 3–6 months for 3 years, then every 6–12 months for 2 years, then annually	Every 12 months
National Comprehensive Cancer Network	USA	1–4 times per year for 5 years, then annually	Every 12 months

As stated, the primary goal of surveillance programmes is to identify recurrence at the earliest stage, thereby enabling early intervention with a subsequent improvement in overall survival.^10,11^ However, trends in how breast cancer recurrences are diagnosed have changed over time. As demonstrated, clinical examination forms part of annual surveillance and previously was the principal method of detecting recurrence.^16^ However, more recent evidence has suggested that radiological surveillance is not only more effective in detecting recurrence, but unsurprisingly does so at an earlier stage,^16^ with a direct effect on survival in such patients.^11^ Conversely, recurrence diagnosed by clinical examination is associated with poorer longer term oncological outcomes.^16–18^ Despite these results, clinical review is still recommended in many countries for at least 5 years, with 70% of in-breast recurrences presenting within 5 years of treatment.^19,20^ The role of routine clinical examination assessing asymptomatic patients with a history of breast cancer as part of a follow-up programme remains a topic of debate, as well as representing a significant financial burden and use of time for busy clinicians. It is likely that such programmes could be optimised.

The aim of this study was to assess how breast cancer recurrences are detected, with a particular focus on the role of routine clinical examination. The results of this study can have important implications on optimal follow-up strategies in women diagnosed with breast cancer to identify recurrent or a new breast cancer.

## Patients and Methods

### Patient Inclusion

A retrospective review of a prospectively held database from a symptomatic breast cancer service in a tertiary referral academic teaching hospital was performed. All patients who were diagnosed between the years 2010 and 2020 with a local breast cancer recurrence or a second primary breast cancer were included. All patients were over the age of 18 and recurrence was defined as histologically proven disease in the same breast, ipsilateral chest wall, local skin region, or in regional lymph nodes. Early recurrence was defined as recurrence within 12 months of original diagnosis with late recurrences being defined as those occurring beyond 12 months of initial diagnosis. A second primary was defined as being distant to the original site in the ipsilateral breast or in the contralateral breast and confirmed histologically. This was considered to be equivalent to a locoregional recurrence as it was often not possible to distinguish between the two diagnoses and the presentation was similar. Patients were excluded where their data was incomplete or where they had evidence of distant metastatic disease. The study was approved by the local hospital audit committee and was performed in accordance with STROBE guidelines.

### Surveillance Process after a Cancer Diagnosis

Following a diagnosis of a primary breast cancer, the following follow-up protocol is employed in our institution as per national guidelines:

- Annual mammogram for a minimum of 5 years (or until age 70, whichever is later – NCCP guidelines). Patients who required breast conserving surgery underwent standard mammography, whilst those requiring mastectomy underwent contralateral mammography.
- Years 0–2 post diagnosis, 6-monthly appointments for clinical review (history and examination)
- Years 3–5 post diagnosis, annual appointments for clinical review (history and examination)

Chest wall imaging on the affected side in those who had undergone mastectomy was only performed when a clinical symptom/sign was detected by the patient or clinician.

### Data Collected

Patients within the database were added after multidisciplinary team discussion and coding. Patient notes, in-hospital digital records and the hospital’s histopathological database were accessed to collect clinicopathological data (including tumour subtype/grade/receptor subtype and size). The national integrated medical imaging system NIMIS was also used to ensure all radiological data was complete. Date of diagnosis, time since original cancer diagnosis and treatment types, including surgical intervention and adjuvant therapies, were also recorded for each patient. Radiological abnormalities were classified using the BI–RADS classification and the reason for imaging (planned surveillance versus investigation of new symptom/sign) was also recorded.^21,22^

### Outcomes

The primary outcome was the mode in which the recurrence or second breast cancer was diagnosed. The total number or recurrences and mode of recurrence was recorded. The modes of recurrence were:

- Self-detection by the patient
- Detection on clinical examination during routine clinical assessment
- Detection on imaging [mammogram (MG) and/or ultrasound (US), computed tomography (CT) or magnetic resonance imaging (MRI)]

### Statistical Analysis

Recurrences were separated by mode of recurrence detection. Duration of time from primary diagnosis to recurrence was presented as median with ranges. Differences in detection mode based on time from primary breast cancer diagnosis were assessed using chi-squared test. A *p* value of less than 0.05 was considered statistically significant.

## Results

A total of 233 patients were identified as having a recurrence or new breast primary from 2010 to 2020. Following application of exclusion criteria, a total of 140 patients were included. The median age at primary diagnosis was 51 years (range 31–87 years) overall. Radiologically diagnosed patient’s primary diagnosis median age was 50 (range 31–84 years), with clinically diagnosed patient’s median age 56 (range 33–87 years). The median age at recurrence or second primary diagnosis was 58 years (range 34–91 years) overall. Recurrence or second primary radiologically diagnosed patient’s median age was 54 (range 37–86 years), with clinically diagnosed patient’s median age 61 (range 36–91).

### Clinicopathological Characteristics

Data on tumour histological subtypes, receptor subtype, tumour size and surgical procedures for both the primary and recurrent breast cancers are shown in Table 2. The median total tumour size (initial primary–invasive and in situ) across the entire cohort was 35.5 mm (range 3–150 mm). Median total (invasive and in situ) tumour size was 35 mm (range 3–10 mm) in the radiologically diagnosed group and 42 mm (range 8–150 mm) in the clinically diagnosed group (*t*-test; *p* = 0.01). Median invasive (only) tumour size was 21 mm (range 2–110 mm) in the radiologically diagnosed group and 26 mm (range 2–170 mm) in the clinically diagnosed group (*t*-test; *p* = 0.03). The median recurrent invasive tumour size was 12 mm (range 2–150 mm). The median recurrent tumour size in the radiologically detected group was 8 mm (range 2–150 mm) compared with 47.5 mm (range 10–115 mm) in the clinically detected group (*t*-test; *p* = 0.001).

**Table 2:** Clinicopathological characteristics of patients at time of initial presentation and time of recurrence

	Initial Primary–*N* (%)	Recurrence–*N* (%)
*Tumour subtype*		
Ductal	82 (58.6%)	83 (59.3%)
DCIS only	18 (12.9%)	7 (5%)
Lobular	13 (9.3%)	12 (8.6%)
Tubular	3 (2.1%)	0 (0%)
Mixed	2 (1.4%)	0 (0%)
Other/unknown	23 (16.4%)	31 (22.1%)
*Oestrogen receptor status*		
Positive	86 (61.4%)	83 (59.3%)
Negative	26 (18.6%)	57 (40.7%)
Unknown	28 (20%)	0 (0%)
*Progesterone receptor status*		
Positive	58 (41.4%)	47 (33.6%)
Negative	54 (38.6%)	93 (66.4%)
Unknown	28 (20%)	0 (0%)
*HER2 receptor status*		
Positive	20 (14.3%)	18 (12.9%)
Negative	92 (65.7%)	118 (87.1%)
Unknown	28 (20%)	0 (0%)
Tumour size	35.5 mm (range 3–150 mm)	12 mm (range 2–150 mm)
*Breast surgery type*		
BCS	64 (45.7%)	21 (15%)
Mastectomy	62 (44.3%)	43 (30.7%)
Local excision	0 (0%)	62 (44.3%)
Unknown	14 (10%)	0 (0%)
*Breast surgery type*		
SLNB	57 (40.7%)	44 (31.4%)
ALND	48 (34.3%)	20 (14.3%)
No axillary procedure	35 (25%)	76 (54.3%)

### Diagnosis of Recurrence

The results revealed that 75/140 (53.6%) patients with a history of breast cancer were found to have abnormalities radiologically leading to a diagnosis of recurrence or second breast primary, while 65/140 (46.4%) were found to have clinically detected abnormalities which led to a diagnosis of recurrence or second primary.

Of those diagnosed clinically, 59/65 (90.7%) presented to the breast clinic with a symptom that was self-detected. We observed that 4/65 (6.2%) patients had underlying cognitive impairment and had an abnormality detected by a carer or next-of-kin. Moreover, 2/65 (3.1%) of patients were found to have abnormalities on annual planned clinical examination by a clinician in the breast clinic (scheduled surveillance) that led to a diagnosis of recurrence. The most common symptoms that patients presented to the breast clinic with (outside of their scheduled appointments) were palpable breast lumps (28%), axillary lumps (20%), chest wall/incision lumps (in case of previous mastectomy, 9%), abscess formation (2%), cutaneous erythema/thickening/puckering (7%) and nipple retraction (3%), as shown in Table 3.

**Table 3:** Mode of diagnosis and time to recurrence

Clinical		Radiological	
Most common presenting symptom:	*N* (%)	Most common modality identifying abnormality:	*N* (%)
Palpable breast lump	18 (28%)		
Axillary lump	13 (20%)	Mammogram	50 (67%)
Chest wall lump (in cases of mastectomy)	6 (9%)	Computed tomography (performed for other indication)	19 (25%)
Wound/mastectomy bed change	13 (20%)		4 (5%)
Skin change (erythema/thickening/retraction)	4 (7%)	Magnetic resonance imaging ultrasonography	2 (3%)
Swelling	3 (5%)		
Mastalgia (only)	2 (3%)		
Mastitis/abscess	1 (2%)		
Nipple abnormality	2 (3%)		

Median time to recurrence:	37 months (range 2–231 months)	Median time to recurrence:	51 months (range 2–263 months)
% of recurrences occurring within 1 year of primary surgery	10.25% (7/65)	% of recurrences occurring within 2 years of primary surgery	6.67% (5/75)
% of recurrences occurring within 5 years of primary surgery	44.6% (29/65)	% of recurrences occurring within 5 years of primary surgery	40% (30/75)

A total of 75/140 (53.6%) of recurrences were diagnosed radiologically. These were diagnosed by mammography (MG) (67%), ultrasound (5%), magnetic resonance imagery (MRI) (3%) and computerised tomography (CT) (25%). The breakdown of diagnosis by imaging modality is shown in Table 3.

### Time to Recurrence

Time to recurrence (months) for both groups was recorded. Overall median time to recurrence was 48 months (range 2–263 months). Median time to recurrence in the clinically detected group was 37 months (range 2–231 months), whilst the median time to recurrence in the radiologically detected group was 51 months (range 2–263 months); *t*-test, *p* = 0.09. Patients diagnosed with recurrence greater than 5 years (outside of routine scheduled clinical exam) after initial diagnosis were excluded, the median overall time to recurrence was 26 months (range 2–60 months); clinical and radiological time to recurrence were 23 months (range 2–58 months) and 33 months (range 2–60 months), respectively; *t*-test, *p* = 0.1, as shown in Table 3. A total of 12 patients experienced an early recurrence within 12 months of their surgery.

### Surgical Technique

A total of 64 patients had undergone breast conserving surgery (wide local excision) for their primary tumour, whilst 62 had undergone mastectomy. Of the 64 patients who had initial breast conserving surgery, 43 proceeded to have a mastectomy upon diagnosis of recurrent disease. The incidence of recurrences or new primaries amongst those who had undergone wide local excision (WLE) versus mastectomy (MTX) at initial operation was also analysed. The median interval at which recurrence or new primaries developed in patients who had an initially undergone WLE was 64 months (range 4–263 months) compared with 27 months (range 2–231 months) in those who had initially had a mastectomy (*t*-test; *p* < 0.01). In the clinically detected group, patients who had a recurrence or new primary who had initially undergone WLE were diagnosed at a median interval of 79 months (range 13–135 months) compared with a median interval of 26 months (range 2–231 months) in those who had initially undergone a mastectomy (*t*-test; *p* < 0.01). In the radiologically detected group, patients who had a recurrence or new primary who had initially undergone WLE were diagnosed at a median interval of 64 months (range 4–263 months) compared with a median interval of 28 months (range 2–76 months) in those who had initially undergone a mastectomy (*t*-test; *p* < 0.01).

## Discussion

The current study demonstrates that routine scheduled annual clinical follow-up appears to add little value in the detection of breast cancer recurrence or new primary detection. Close radiological surveillance, as well as a standardised pathway in which patients with a history of breast cancer with new breast symptoms can be seen in a timely manner, is likely to identify the majority of local breast cancer recurrences or new primary. The study also demonstrates that whilst surgical and non-surgical treatments for breast cancer have become more personalised over the last decade, surveillance programmes remain generalised with little focus on identifying patients who are at higher risk for recurrence and therefore require close surveillance. It is likely that there is still a role for assessing patients deemed to be higher risk on a scheduled clinical basis annually, but this study demonstrates that many patients do not require scheduled annual outpatient visits, as is the case currently. It is estimated that the institution in which the study was performed schedules close to 5000 “follow-up” appointments annually, placing a huge economic, time and financial burden on specialist staff who oversee this programme.

Although routine annual clinical surveillance is recommended in Europe^13^ and the USA,^23^ there remains little evidence to support its function with regard to the detection of local recurrence or distant metastatic disease.^16,24,25^ While overall survival and recurrence rates in breast cancer have improved over the last decade,^26,27^ largely due to improving adjuvant therapies and a stronger focus on obtaining negative margins intra-operatively, there remains debate as to the duration of surveillance for patients with breast cancer. Previous studies have suggested that patients who have undergone breast-conserving surgery (BCS) experience local recurrences most frequently 4 years after surgery, compared with patients who have undergone mastectomy, in which recurrences are seen most commonly at 2 years.^28^ Equally, for patients with ER+ disease, the risk of local recurrence can persist beyond 10 years after surgery.^29^ Few patients will experience a true recurrence within the first 12 months after surgery. Our study adds to those findings, demonstrating that such recurrences were most common more than 5 years after surgical treatment in those undergoing BCS. For patients who had undergone mastectomy, recurrences were seen within 2–3 years. The use of mammography as the primary surveillance tool as opposed to annual clinical exam is particularly pertinent to BCS patients, as 75% of recurrences are within the ipsilateral remnant breast (which mammogram assesses) with only 5–15% presenting with distant metastatic disease. In addition, the current study demonstrates that the median time at which recurrence occurs in those who have undergone BCS is beyond 5 years, outside the window of scheduled clinical surveillance. In contrast, 30% of patients who have undergone mastectomy and subsequently develop a recurrence will present with distant metastatic disease. Whilst the benefits of radiological surveillance in patients undergoing breast-conserving surgery are clear, there remains debate over the optimal manner in which mastectomy patients should be monitored. A recent meta-analysis by Smith et al. has indicated that there is no benefit to radiological surveillance in mastectomy patients, but that the evidence is limited in this regard.^30^ The diagnosis of recurrent distant metastatic disease is associated with a poor prognosis, and it appears that surveillance either by imaging or annual clinical review has little effect on overall survival.

The findings of this study certainly support the provision of mammography as the primary surveillance tool in breast cancer survivors, as only 2 patients out of 81 who were diagnosed through clinical exam had a normal mammogram. Conversely, over half of recurrences within the study period were detected through annual mammography. Separately, the need for ongoing annual mammography and the age at which radiological surveillance should cease remain controversial. Other national guidelines, such as those published by the National Institute of Clinical Excellence in the UK, have argued that as the risk of recurrence in many patients falls over time to risk levels in line with unaffected females, many of these breast cancer survivors could be managed under the auspices of the national breast screening programme.

Although previous evidence has demonstrated that clinical examination has a lower sensitivity^25^ when compared with mammography for the diagnosis of locoregional recurrence, there remains a group of patients that present to the breast clinic with new symptoms outside of their scheduled radiological/clinical surveillance programme. The majority (*n* = 57) of the 81 patients diagnosed clinically presented with having noticed the symptom(s) themselves. This is in keeping with published evidence demonstrating that 80% or more patients with breast cancer will identify locoregional recurrence themselves outside of scheduled clinical surveillance.^31–33^ Previous studies have also demonstrated that breast cancer survivors are increasingly likely to take full personal responsibility for the investigation of new symptoms after treatment.^34,35^ As a result of this finding and of the increasing evidence that demonstrates the minimal value of routine clinical follow-up, many centres in the UK now offer annual mammography as the main surveillance tool to patients. In addition, patients are frequently enrolled in an open access follow-up programme,^15^ enabling them to receive rapid access to the unit in which they were treated if they develop new symptoms. They are then seen urgently by a breast surgeon or oncologist who can expedite radiological and/or histological investigation. Patient education can also play a key role in informing patients of what to look for and when to seek the appropriate assessment to investigate new symptoms. Such a system has not been assessed in randomised trials, however, and as a result many of the guidelines still advocate regular clinical review.

It is likely that there will remain a cohort of patients who will continue to benefit from clinical surveillance going forward. These patients include those who are deemed to be “high risk” on account of younger age, triple negative subtype, or those who require alternate surveillance strategies such as annual MRI scans. A previous study by Wirtz et al.^36^ indicates that younger patients are less likely to attend for surveillance imaging compared with their older counterparts, suggesting that a different surveillance programme may be beneficial in this group. In addition, there are patients who struggle after a breast cancer diagnosis and, for psychological reasons or otherwise, find it challenging to self-examine or present in the setting of new symptoms. In these patients, the role of other modalities such as circulating tumour DNA or miRNA obtained by liquid biopsy, which have shown significant promise and are the subject of ongoing prospective studies, may also become more prominent within surveillance programmes for breast cancer, especially in younger patients with triple-negative disease.^37–40^

The increasing role of survivorship programmes in such patients who have undergone breast cancer treatment is of particular importance. Survivorship is no longer focussed solely on monitoring for recurrence, but now encompasses a number of other factors that includes the management of both physical and psychological effects of treatment, as well as adherence to adjuvant treatments such as tamoxifen.^41^ It is plausible that the management of survivorship should take place in an alternative setting where physicians (such as primary care providers) or nurses are better equipped to deal with the range of challenges that patients with cancer now face, but with the option of easily accessing specialist breast input as needed.^42,43^ Previous evidence has also demonstrated that attending clinics for follow-up can increase anxiety levels in patients, and that the use for the example of a nurse-led telephone follow-up clinic can minimise anxiety, as well as building a trusting relationship which patients value.

The study has a number of limitations. Whilst the focus of the study was on patients with a history of breast cancer who developed recurrence or second primary, as well as the management of such lesions, details on longer term survival in such patients is not reported. Such outcomes are unlikely to influence the pertinent study result, demonstrating that scheduled clinical examination adds little insight. The study is also retrospective. Prospective data with long-term follow-up of patients diagnosed with recurrence with a focus on identifying patients who are high-risk and who may require clinical follow-up on a scheduled basis is needed. It is possible that a combination of patient characteristics, such as age and gene status, clinicopathological characteristics such as tumour grade or size, as well as multigene signature panels, could be used to identify patients who require closer surveillance or those who require follow-up beyond 5 years.

## Conclusion

The current study demonstrates that less than 5% of patients with breast cancer recurrence are diagnosed at routine clinical examination as part of a scheduled breast cancer clinical surveillance programme. It is likely that annual clinical examination provides little value in the diagnosis of recurrence, and surveillance programmes may benefit from reduced focus on such a modality in diagnosing recurrent breast cancer with potential benefits on surgeon workload. More focus should be placed on regular appropriate radiological assessment, as well as ensuring patients have urgent and ease of access to a breast clinic if they develop new symptoms/signs. There may be a role for clinical surveillance in higher risk patients, but such programmes require further investigation.
